# Alterations to Titanium Surface Depending on the Fluorides and Abrasives in Toothpaste

**DOI:** 10.3390/ma15010051

**Published:** 2021-12-22

**Authors:** Takahiro Shuto, Yuichi Mine, Seicho Makihira, Hiroki Nikawa, Takanori Wachi, Kazutoshi Kakimoto

**Affiliations:** 1 Department of Oral Health Engineering, Faculty of Health Sciences, Osaka Dental University, 1-4-4 Makinohonmachi, Osaka 573-1144, Japan; kakimoto@cc.osaka-dent.ac.jp; 2 Department of Medical System Engineering, Division of Oral Health Sciences, Graduate School of Biomedical and Health Sciences, Hiroshima University, 1-2-3 Kasumi Minami-ku, Hiroshima 734-8553, Japan; mine@hiroshima-u.ac.jp; 3 Department of Oral Biology & Engineering, Division of Oral Health Sciences, Graduate School of Biomedical and Health Sciences, Hiroshima University, 1-2-3 Kasumi Minami-ku, Hiroshima 734-8553, Japan; spypondfamily@icloud.com (S.M.); hirocky@hiroshima-u.ac.jp (H.N.); 4 Section of Fixed Prosthodontics, Division of Oral Rehabilitation, Faculty of Dental Science, Kyushu University, 3-1-1 Maidashi, Fukuoka 812-8582, Japan; wachi@dent.kyushu-u.ac.jp

**Keywords:** titanium surface, toothpaste, brushing, fluoride, abrasive, corrosion

## Abstract

Fluoride and abrasives in toothpastes may cause corrosion and deterioration of the titanium used for implants and other prostheses. The purpose of this study was to investigate how the presence or absence and types of fluoride and abrasives affected the titanium surface texture. Brushing with toothpastes was performed on pure-titanium discs using an abrasive testing machine. Unprocessed titanium discs without brushing were used as control samples. Surface roughness, color, and gloss of titanium were measured and the differences compared with the control were analyzed. Additionally, titanium surfaces and abrasives in toothpastes were observed using a scanning electron microscope to compare the surface texture of each sample. Some toothpastes (abrasive+) significantly increased the difference in surface roughness, color, and gloss, compared with ultrapure water. Toothpaste (fluoride+/abrasive+) that had many polygonal abrasive particles led to the largest color differences and exhibited notable scratches and a larger number of contaminant- or corrosion-like black spots. In contrast, brushing with toothpaste without fluoride or abrasives (fluoride−/abrasive−) caused little change to the titanium surface. These results suggest that both fluoride and abrasives in toothpaste used for brushing may be factors that affect surface texture and corrosion resistance of titanium.

## 1. Introduction

Titanium generates a strong surface-oxide film (TiO_2_) to enhance its corrosion resistance and achieve superior biocompatibility [1]. In addition, titanium’s mechanical properties enable its broad use as a dental material in oral cavities for applications such as dental implants, orthodontic appliance wires, and other prostheses [2,3,4,5].

However, several studies have reported that the corrosion resistance of titanium decreases in the presence of fluorine [6,7,8,9]. Moreover, fundamental research on the corrosion resistance of titanium under various pH levels in the presence of fluorides has demonstrated that the fluoride-containing solutions and pastes may corrode the titanium used for dental implants in acidic conditions [10,11,12,13]. Nevertheless, most of the commercially available toothpastes contain fluoride for caries prevention [14]. In Japan, Europe, and the United States, fluorides such as sodium fluoride (NaF), sodium monofluorophosphate (MFP), and tin fluoride (SnF_2_) have been approved for use in commercial toothpastes [15]. According to ISO standards, the maximum fluoride content in toothpaste is 1,500 ppm [15]. In context, a clinical trial on adults and the elderly established that fluoride toothpaste practically prevents caries [16]. As such, numerous individuals with titanium implants or prostheses in the oral cavity regularly use toothpastes containing fluoride.

In context, brushing or abrasive testing with toothpaste containing abrasives can cause surface abrasion on titanium materials, and even mechanical stimulation with purified water has been found to cause damage and create unevenness on titanium surfaces [17,18,19,20]. In the human oral cavity, wear mechanisms such as two-body, three-body, fatigue, and corrosive wear occur; the two-body wear mechanism is caused by the direct contact of two materials, while in the three-body wear mechanism, an abrasive medium is added as a wear factor [21,22]. These studies indicate that daily brushing could cause titanium abrasion in the oral cavity, and we hypothesized that both fluorides and abrasives in the toothpaste are associated with variations in the surface properties of titanium.

Thus, this study aimed to investigate the influences of fluoride- and abrasives-containing toothpastes on titanium surface texture. Consequently, a brushing test was performed using various commercial toothpastes, including products developed and marketed for implants that contain neither fluorides nor abrasives. Furthermore, we examined the influence of the presence or absence of fluoride and abrasives, as well as the types of fluoride and abrasives, on the surface roughness, color, and gloss of titanium.

## 2. Materials and Methods

### 2.1. Materials

The four types of commercial toothpaste used in this study are presented with corresponding abbreviations in Table 1. These toothpastes were classified according to their contents as follows: both fluoride and abrasive (fluoride+/abrasive+), fluoride without abrasive (fluoride+/abrasive−), abrasive without fluoride (fluoride−/abrasive+), and neither fluoride nor abrasive (fluoride−/abrasive−). Two different toothpastes were used for each type. The fluoride content of each toothpaste was determined from the product information, and the pH of the toothpastes and slurries were measured using a pH meter (ISPET pH meter KS723; BAS Inc., Tokyo, Japan). The toothbrush used in the abrasion test was Butler #211 (Sunstar Inc., Osaka, Japan). Moreover, 60 mirror-polished pure titanium discs (JIS2, 15 mm diameter, 1 mm thickness, Oda Kouki Co., Ltd., Osaka, Japan) that were manufactured through cold rolling were used as the titanium specimen in this study.

### 2.2. Slurry Preparation and Toothbrush Abrasion Test

According to the ISO standards (TS14569-1), the toothpaste slurry was prepared at a ratio of 1 g of toothpaste to 2 ml of ultrapure water (MQ) [23]. The action of brushing was performed using an abrasion testing machine (K760; Tokyo Giken Inc., Tokyo, Japan). Following ISO standards (TS14569-1) [23] and a report by Hossain et al. [24], 350,400 brushstrokes were performed with a stroke length of 10 mm at 120 rpm under a load of 2.5 N. The toothbrush was replaced after completing half of the brushing, or 175,200 strokes. In addition, the titanium discs were brushed with MQ, and an unprocessed titanium disc that was not brushed was used as control. Titanium disc specimens were allocated to a total of 10 groups of six specimens each.

### 2.3. Titanium Surface Analysis

After the abrasion tests, the titanium discs were rinsed with MQ and subjected to ultrasonic cleaning in acetone and ethanol for 15 min each. After drying, the surface roughness, color, and gloss of the titanium discs were measured and analyzed.

The surface roughness was measured using a surface roughness meter (Surfcorder SE3300; Kosaka Laboratory Ltd., Tokyo, Japan) at five arbitrarily selected points on each titanium disc, and the mean value was adopted as the representative surface roughness value of the sample. Consequently, the variation in the surface roughness before and after the abrasion tests was evaluated using the representative values of these samples.

In addition, the color differences were analyzed based on color values obtained using a color difference meter (NF333; Nippon Denshoku Industries Co., Ltd., Tokyo, Japan), wherein the L*, a*, and b* values were measured at five arbitrarily selected points on each titanium disc, and the mean value was adopted as the representative color value of the sample. In particular, the color values obtained before (L^1^*, a^1^*, b^1^*) and after (L^2^*, a^2^*, b^2^*) the test were used to evaluate the color differences (⊿L = L^2^* − L^1^*; ⊿a = a^2^* − a^1^*; ⊿b = b^2^* – b^1^*), which were subsequently used in the following formula: ⊿Eab = [(⊿L)^2^ + (⊿a)^2^ + (⊿b)^2^]^1/2^.

Moreover, the gloss value was measured using a glossmeter (PG-II M; Nippon Denshoku Industries Co., Ltd., Tokyo, Japan) at five arbitrarily selected points on each titanium disc, and the mean value was adopted as the representative gloss value of the sample. The variations in gloss values before and after the abrasion tests were compared using the representative values of these samples. The gloss value obtained when shining incident light at 60° at a 10% specular reflection rate onto a glass surface with a refractive index of 1.567 was considered as 100.

### 2.4. Scanning Electron Microscopy Observation

Scanning electron microscopy (SEM) equipment (VE-8800; Keyence Corp., Osaka, Japan) was used to observe the titanium disc surfaces for comparing the surface textures of the samples (original magnification of 500×).

Following the method of Tsuruta et al. [25], the toothpaste slurries were centrifuged at 10,000 rpm for 20 min, after which the sediment was isolated, homogenized, and centrifuged with 100% ethanol. Subsequently, this sediment was dried to observe the abrasive particles in the toothpaste.

### 2.5. Data Analysis

The deviation in the mean values of groups were subjected to one-way analysis of variance (ANOVA) and Tukey’s multiple range test. In addition, *p* < 0.05 and *p* < 0.01 were considered to indicate statistical significance.

## 3. Results

### 3.1. Observation of Abrasives in Toothpaste

**Figure 1:**
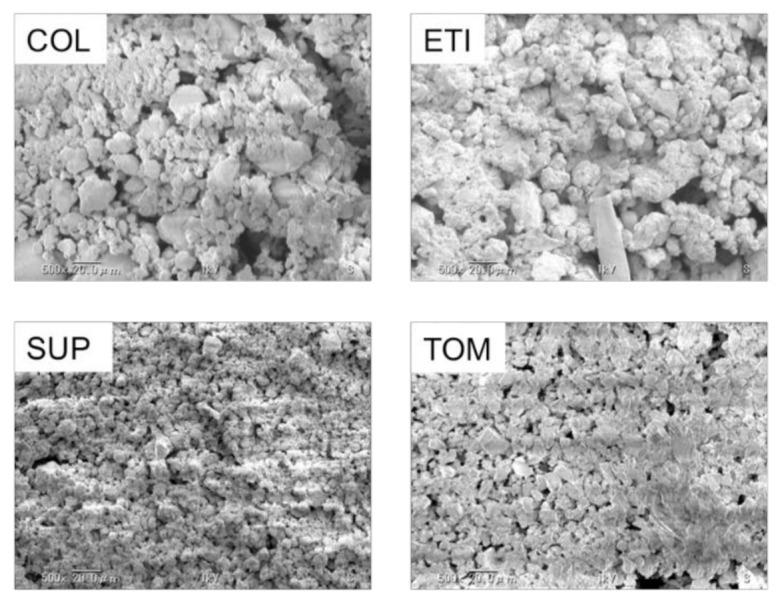
SEM images of abrasive particles in toothpastes, magnification of 500×.

The SEM observations illustrated the presence of a large number of small spherical particles along with a small number of large polygonal particles in COL (fluoride+/abrasive+) and ETI (fluoride−/abrasive+), which contain only silica as abrasives. On the other hand, SUP (fluoride+/abrasive+) and TOM (fluoride−/abrasive+) contain heavy calcium carbonate and displayed numerous uniformly sized polygonal particles. Moreover, the abrasive densities of SUP and TOM were greater than those of COL and ETI (Figure 1).

### 3.2. Variations in Titanium Surface Roughness after Brushing with Toothpastes

**Figure 2:**
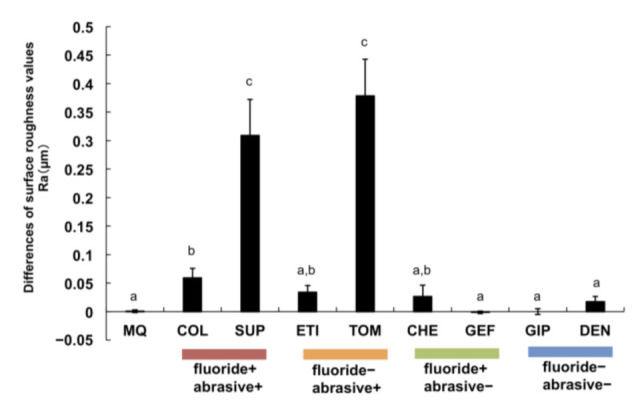
Influence of brushing with toothpastes or MQ on titanium surface roughness. Surface roughness values represent the variations with the values of the control sample. Independent experiments were repeated three times. Data represent the means ± SD of triplicate experiments. The different letters among the samples indicate a statistically significant difference (*p* < 0.05 or *p* < 0.01).

The surface roughness was measured and the variations compared to the control values were evaluated. The surface roughness of the titanium discs brushed with all abrasive+ toothpastes was greater than those brushed with MQ. In particular, the variations in surface roughness with COL, SUP, and TOM (abrasive+) were significantly greater than those with MQ (*p* < 0.05). Furthermore, the variations in surface roughness with SUP and TOM were remarkably greater than those with all other toothpastes, including abrasive+ toothpastes (*p* < 0.01, Figure 2). The surface roughness of samples after brushing with toothpastes, such as CHE, GEF, GIP, and DEN (abrasive–), did not vary from those brushed with MQ.

### 3.3. Alterations in Titanium Color after Brushing with Toothpastes

**Figure 3:**
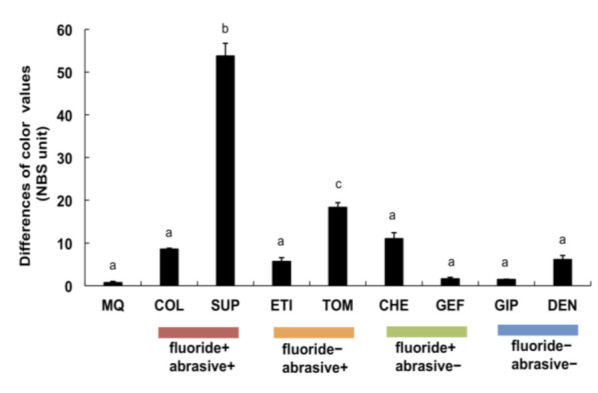
Influence of brushing with toothpastes or MQ on titanium color. The color values represent the variations with the values of control sample. Independent experiments were repeated three times. Data represent the means ± SD of triplicate experiments. The different letters among the samples indicate a statistically significant difference (*p* < 0.05 or *p* < 0.01).

The color values were measured, and their variations with the control were calculated accordingly. The color differences between the SUP (fluoride+/abrasive+) and TOM (fluoride–/abrasive+) were significantly greater than those obtained after brushing with MQ and other toothpastes (*p* < 0.05). Specifically, brushing with SUP (fluoride+/abrasive+) caused the largest color variation among all the toothpastes (*p* < 0.01, Figure 3). However, all the toothpastes without abrasives did not vary in color differences from MQ.

### 3.4. Variations in Titanium Gloss after Brushing with Toothpastes

**Figure 4:**
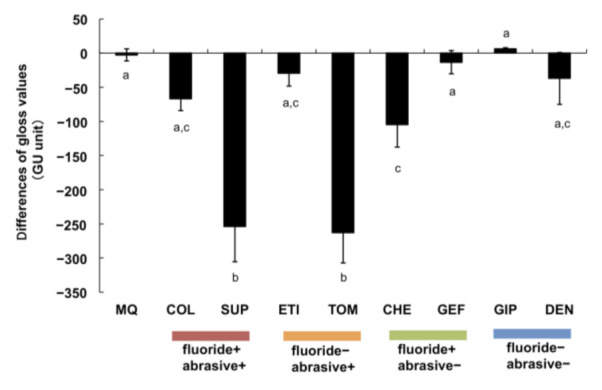
Influence of brushing with toothpastes or MQ on titanium gloss. The gloss values represent the variations with the values of the control sample. Independent experiments were repeated three times. Data represent the means ± SD of triplicate experiments. The different letters among the samples indicate a statistically significant difference (*p* < 0.01).

The gloss values were measured, and their variations from the control values were calculated. The gloss values obtained with SUP (fluoride+/abrasive+) and TOM (fluoride–/abrasive+) were significantly lower than those obtained with MQ and the other toothpastes (*p* < 0.01). In addition, the gloss value with CHE (fluoride+/abrasive–) was significantly lower than that with MQ (*p* < 0.01, Figure 4).

### 3.5. Titanium Surface Observations

**Figure 5:**
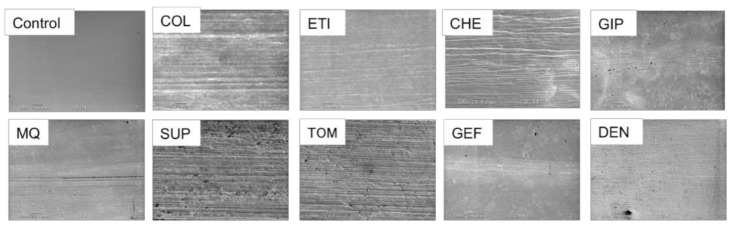
SEM images of titanium disc surfaces brushed with MQ or toothpastes and an unprocessed titanium disc (control), magnification of 500×.

Macroscopically, the surfaces of all the brushed titanium discs exhibited scratches from brushing, and a number of them displayed alterations in factors of color and gloss (data not included). Furthermore, detailed observations with SEM exhibited deep grooves, surface coarsening, and a larger number of contaminant- or corrosion-like black spots on the titanium surfaces, especially those brushed with SUP (fluoride+/abrasive+) and TOM (fluoride−/abrasive+). In contrast, titanium discs brushed with MQ or abrasive– toothpastes, such as GEF, GIP, and DEN, exhibited thin and shallow grooves in comparison to those brushed with abrasive+ toothpastes (Figure 5).

## 4. Discussion

Toothpaste, mouth-rinse solutions, and tooth surface embrocations are commonly used for oral care and hygiene. Many of these products contain fluoride, as fluorine is widely known to prevent caries in natural teeth [14,26,27,28]. However, the use of fluoride-containing products is unfavorable for patients with titanium implants or other titanium prostheses owing to their corrosive effects against titanium under high fluorine concentrations or acidic conditions [6,10,11,29]. There have been a number of studies on the relevance of fluoride concentration and pH to titanium corrosion resistance [10,30,31,32,33], and Kimura et al. [11] presented the safety and risk range of corrosion. The toothpastes used in the present study were general-use products, so their fluoride concentration and pH were within the safe range (Table 1). Apart from fluoride, toothpastes contain abrasives as one of the components. The abrasives consist mainly of water-insoluble inorganic powder, such as silica, heavy calcium carbonate, zeolite, calcium hydrogen phosphate dihydrate, calcium hydrogen phosphate, hydroxyapatite, and aluminum hydroxide.

In context, several studies have been conducted to characterize the influences of fluoride or brushing on titanium. We demonstrated that the presence or absence of fluoride in toothpaste (950–1000 ppm, pH 6.2–8.4) has no impact on the color and roughness of titanium surfaces without brushing in vitro [34]. However, Gomi et al. [35] reported that toothpaste containing fluoride (950 ppm, pH 7.7) invaded titanium materials in an immersion experiment. Moreover, Fais et al. [17] conducted both immersion and toothbrush abrasion experiments and observed that the titanium surfaces were not invaded upon in the immersion group, but were in the group of brushing with toothpaste containing or not containing fluoride (1500 ppm, pH 6.3). Acharya et al. [19] proposes that the influences of brushing with toothpaste containing fluoride on the titanium surface are due to a reaction of the fluoride ions with the surface oxides on the titanium, whose protective properties are remarkably reduced. Brushing experiments include a study by Hossain et al. [24], which focused on abrasives and found that a titanium surface was abraded and roughened depending on the abrasives in toothpastes without fluoride. They found that the interaction between the titanium surface and abrasives is pH dependent [36]. However, Faria et al. [37] showed that even though toothpaste with both alkaline and acidic pH increased roughness after brushing, there was no significant difference.

Although there is disagreement between the findings of basic studies owing to varying experimental conditions, these studies suggest that the degradation and corrosion effects of fluoride against titanium and the physical invasion by abrasives accelerate the alterations occurring in the titanium surface texture. Acharya et al. [19] conducted in vitro experiments similar to those in this study, comparing the effects of brushing with medium abrasive toothpaste (fluoride+) between different materials, and concluded that titanium is more susceptible to corrosion and abrasion, which seems to support our hypothesis. Furthermore, in terms of brushing experiments, previous studies have limited the types of toothpaste compared. Thus, we utilized a variety of toothpastes in the present study to verify these findings.

Consequently, we determined that brushing with toothpaste caused varying degrees of abrasion on titanium surfaces, and abrasive+ toothpastes accelerated the abrasion of these surfaces. In contrast, the degree of abrasion caused by abrasive– toothpastes was similar to that obtained with MQ (Figure 2). Similar findings were observed with color and gloss variations on the titanium surface, i.e., brushing with abrasive– toothpastes exhibited similar effects to brushing with MQ, whereas SUP and TOM (abrasive+) prominently influenced the color and gloss of titanium surfaces (Figure 3 and Figure 4). These results are potential indicators of titanium surface alterations and were also confirmed with surface observations using SEM (Figure 5). Thus, the abrasive+ toothpastes, especially SUP and TOM, caused severe physical invasion of titanium surfaces.

A larger particle size, higher blending amount, and greater Mohs hardness of abrasive particles in toothpaste generally result in greater polishing efficiency; however, abrasives are most likely the source of the physical invasion in titanium. Although there were certain variations in the particle size, the SUP and TOM samples, both containing heavy calcium carbonate which has a Mohs hardness of 5, exhibited relatively uniformly small sized polygonal particles with high density (Figure 1). In contrast, COL and ETI primarily contain silica, which has a Mohs hardness of 7, along with numerous spherical particles and a small proportion of polygonal particles. In addition, they exhibited nonuniform sizes. Overall, although Hossain et al. [24] suggested that the impact on titanium surface texture is principally due to the difference in shape and size of abrasives, our findings indicated that the blending amount or the abrasive type—depending on particle shape and size—pose varying influences on the titanium surface abrasion. In abrasive+ toothpastes, the degree of abrasion may vary with the size uniformity, shape, and density of the abrasive particles rather than particle size and hardness.

In comparison to other toothpastes, the SUP and TOM more strongly impacted the color of the titanium surfaces, possibly because of the surface dullness (data not included) and large number of contaminant- or corrosion-like black spots (Figure 3 and Figure 5). Moreover, hardly any black spots were present on the titanium surfaces brushed with other toothpastes. The toothpastes used in the present study contained 950–1450 ppm of fluoride and displayed a pH of 6.4–8.4 and a slurry pH of 6.8–8.8 (Table 1), all of which were within the safety ranges of the corrosion. Nevertheless, under the experimental conditions of this study, brushing with a specific toothpaste containing both fluoride and abrasives degraded and corroded the titanium. These results are largely consistent with our supposition and the results reported by Fais et al. [17,38] and Acharya et al. [19], which suggests that the abrasives physically invaded the titanium surface to disintegrate the oxide film (TiO_2_) during brushing, and thereafter, the fluoride immediately acted upon the titanium surface that was vulnerable to corrosion. In this study, we selected toothpastes that are recommended for patients with titanium implants, as they contain neither fluorides nor abrasives. Overall, these toothpastes (GIP and DEN) had a slight impact on titanium surfaces because they contain neither fluorides nor abrasives and exist in a gel state that may remain between the titanium surface and brush tips for a long period, thereby suppressing physical invasion.

The above-mentioned findings suggest that alterations to titanium surface texture depend on the presence or absence and type of fluorides and abrasives in toothpastes. In particular, toothpastes containing fluorides and abrasives may accelerate the degradation and corrosion of titanium as compared to those without fluorides and/or abrasives. Therefore, the influence of corrosion- and abrasion-deteriorated titanium implants on the surrounding tissue is a critical concern as well [39]. In fact, it has been reported that elution of titanium ions was significantly higher in the peri-implant tissue of patients with peri-implantitis [40]. The titanium ions induce a general inflammatory response with increases in the production of monocyte and macrophage secreted cytokines, and cause the proliferation of CD4+ and CD8+ cells [41]. In addition, titanium ions enhance expression of RANKL, which is a key factor for bone resorption in activated T cells [42]. Moreover, it has been reported previously that eluted titanium ions may synergistically affect the expression of bone resorption-related molecules with *P. gingivali*s-LPS in the tissue surrounding the implant [43]. Berryman et al. [44] noted an involvement of titanium wear particles in the overexpression of RANKL, IL-33, and TGF-β1. Thus, toothpastes without fluorides or abrasives had a slight impact on titanium surface texture, suggesting that these may be effective for maintaining the homeostasis of peri-implant tissue.

It is not possible to define toothpaste selection criteria for patients with titanium implants or other prostheses solely on the basis of the above results and considerations, as it was not within the scope of the present study to conduct multifaceted experiments in consideration of various characteristics in clinical situations, such as the buffering capacity and diluting effect of saliva, and actual brushing methods and pressure. Although it is difficult to carry out in vivo studies because they are time consuming and require a sufficient number of subjects, further experimental research following clinical practice recommendations and elucidation of the mechanisms of titanium surface alterations in detail by microstructural analysis is required.

## 5. Conclusions

The results of this study suggest that the presence or absence of fluorides and abrasives, as well as the type of fluoride and abrasives, in toothpaste are factors impacting the titanium surface texture, and toothpastes that contain both have a greater impact. Moreover, brushing with toothpaste containing neither fluorides nor abrasives caused slight alterations to the titanium surfaces. As implant treatment is now widespread, the success of the treatment relies on the appropriate maintenance of these implants in the future. Therefore, the findings describing the various risks and benefits of each toothpaste in this basic study should be provided to patients with implants as guidance in selecting toothpaste.

## Figures and Tables

**Table 1:** Toothpastes used in the present study.

Products	Manufacturers	Abbreviations	Fluorides	Abrasives	pH	Slurry pH	State
Total 12 h Protection Super Smile	Colgate Cosmetechs	COL SUP	NaF (1450 ppm) MFP (900-999 ppm)	Silicic anhydride Calcium carbonate Magnesium carbonate	6.8 7.9	7.4 8.3	Paste Paste
Etiquette Lion Aa Fluoride-Free Antiplaque &Whitening Toothpaste	Lion Tom’s OF MAINE	ETI TOM	— —	Silicic anhydride Calcium carbonate Silicic anhydride	6.6 7.8	6.8 8.0	Paste Paste
Check-up Gel Gel Coat F	Lion Weltec	CHE GEF	NaF (950 ppm) NaF (950 ppm)	— —	8.2 8.4	8.6 8.8	Gel Gel
Gel Coat IP Dennovate Implant Teethgel	Weltec Hakusui Trading	GIP DEN	— —	— —	7.1 6.4	7.5 6.8	Gel Gel

## Data Availability

Not applicable.
